# Optimizing Uptake of Multimodal Pain Management After Surgery Using the Electronic Health Record

**DOI:** 10.1001/jamasurg.2023.3654

**Published:** 2023-08-23

**Authors:** Tasce Bongiovanni, Elizabeth Lancaster, Matthias Behrends, Li Zhang, Elizabeth Wick, Andrew Auerbach, Mark J. Pletcher

**Affiliations:** 1 Department of Surgery, San Francisco School of Medicine, University of California San Francisco; 2 Department of Anesthesia, San Francisco School of Medicine, University of California San Francisco; 3 Department of Epidemiology and Biostatistics, San Francisco School of Medicine, University of California San Francisco; 4 Division of Hospital Medicine, San Francisco School of Medicine, University of California San Francisco

## Abstract

This quality improvement study evaluates the effect of an electronic health record intervention on multimodal pain management following surgery in 2 randomized clinical trials.

Multimodal pain regimens that include acetaminophen and nonsteroidal antiinflammatory drugs (NSAIDs) may help reduce the use of opioids for pain management in the postoperative period and are increasingly used in enhanced recovery pathways,^1,2^ although uptake has been challenging. We aimed to increase postoperative multimodal use through an electronic health record intervention and optimize the intervention through sequential randomized controlled testing.

## Methods

This study was approved by the University of California San Francisco, and informed consent was waived as this was deemed a minimum-risk study. We embedded a new pain medication order set in the admission orders with the goal of increasing uptake of multimodal data without burdening clinicians with useless extra clicks.^3^ To test different ways of encouraging use, we conducted a series of randomized clinical trials (RCTs),^4^ randomizing at the clinician level. In the first RCT, we made NSAIDs a required choice in the active intervention arm; that is, clinicians had to either order an NSAID or acknowledge that the patient had an NSAID contraindication vs being able to skip past this step (control). In the second RCT (RCT2), NSAIDs were prechecked in the active intervention arm, and clinicians were rerandomized. Of note, acetaminophen was prechecked for all groups throughout both RCTs. Clinicians could uncheck acetaminophen or NSAIDs and bypass pain medication for patients with no more than mild pain.

We conducted an interrupted time series analysis of all adults admitted after a surgical procedure since January 1, 2020, to analyze changes over time in 3 outcomes: ordering of acetaminophen and NSAIDs by the end of the first full hospital day, and opioid prescribing at discharge. Within each period, each group, and overall, we fit a logistic model with time as the only independent variable and plotted the model predictions as the best fit line. We then tested the null hypothesis that the outcome likelihood was equal at the end of the intervention period compared with immediately prior to RCT1 for each outcome.

## Results

Our analysis included 18 143 unique encounters (mean [SD] age, 58.56 [16.45] years; 9038 [49.8%] female) and randomized 1176 unique clinicians. The mean (SD) Charlson score in the overall population was 2.70 (2.31) (Table). Encounters were statistically similar across each period, except for age in which participants in RCT2 were older. Clinicians randomized to an admission encounter were similar in all characteristics, including average admissions, years working at the University of California San Francisco, sex, department, and clinician type.

**Table.** Unique Encounters

Characteristic	No. (%)				*P* value
	Overall	Prelaunch	RCT1	RCT2	
No.	18 143	6007	7330	4806	NA
Age at admission, mean (SD), y	58.56 (16.45)	57.32 (16.35)	58.46 (16.42)	60.26 (16.47)	<.001
Charlson score, mean (SD)	2.70 (2.31)	2.65 (2.24)	2.72 (2.36)	2.75 (2.34)	.06
Gender					
Female	9038 (49.8)	3104 (51.7)	3602 (49.2)	2332 (48.6)	.01
Male	9090 (50.1)	2899 (48.3)	3723 (50.8)	2468 (51.4)	
Nonbinary	8 (0.0)	3 (0.0)	3 (0.0)	2 (0.1)	
Missing	7 (0.0)	1 (0.0)	2 (0.0)	4 (0.1)	
Race and ethnicity^a^					
American Indian or Alaska Native	108 (0.6)	43 (0.7)	49 (0.7)	26 (0.5)	.69
Asian	2302 (12.8)	735 (12.3)	945 (13.0)	625 (13.1)	
Black or African American	1197 (6.6)	402 (6.7)	503 (6.9)	297 (6.2)	
Latinx	2843 (15.9)	958 (16.0)	1153 (15.8)	735 (15.4)	
Multiple races or ethnicities	536 (3.0)	157 (2.6)	190 (2.6)	131 (2.7)	
Native Hawaiian or Other Pacific Islander	88 (0.5)	35 (0.6)	37 (0.5)	21 (0.4)	
Southwest Asian and North African	217 (1.2)	44 (0.9)	99 (1.4)	74 (1.2)	
White	10 344 (57.4)	3509 (58.8)	4250 (58.4)	2783 (58.4)	
Other^b^	380 (2.1)	130 (2.2)	150 (2.1)	105 (2.2)	
Missing	128 (0.7)	38 (0.6)	53 (0.7)	37 (0.7)	

Abbreviations: NA, not applicable; RCT, randomized clinical trial.

^a^ Race and ethnicity data were collected from the electronic health record based on patient-reported data and were reported for the ability for other centers to generalize the data.

^b^ Listed as other in the electronic health record and therefore could not be defined here.

As shown in the Figure, acetaminophen use increased after initiation of the new order set in which it was prechecked (odds ratio/day, 1.00; 95% CI, 1.00-1.00; slope *P* < .001). ). NSAID prescribing during RCT1 was no different for clinicians using the standard order set (25%) compared with clinicians using the order set where NSAID prescribing was a required choice (25%; *P* > .99). During RCT2, NSAID prescribing was substantially higher for clinicians using a prechecked order compared with those using the required choice order set (47% vs 37%, respectively; *P* < .001). Opioid use during RCT2 was more common in the NSAID prechecked group (60% vs 51%, respectively; *P* < .001), but overall, discharge opioid use started declining (odds ratio/day = 1.00; 95% CI, 1.00-1.00; *P* = .01). We found no difference in potential adverse effects of NSAIDs (new gastrointestinal bleed, new acute kidney injury, and death) between the 2 groups in either RCT. Acetaminophen ordering at admission also rose in the mild pain group. We found no differences when analyzing by race and ethnicity.

**Figure.**
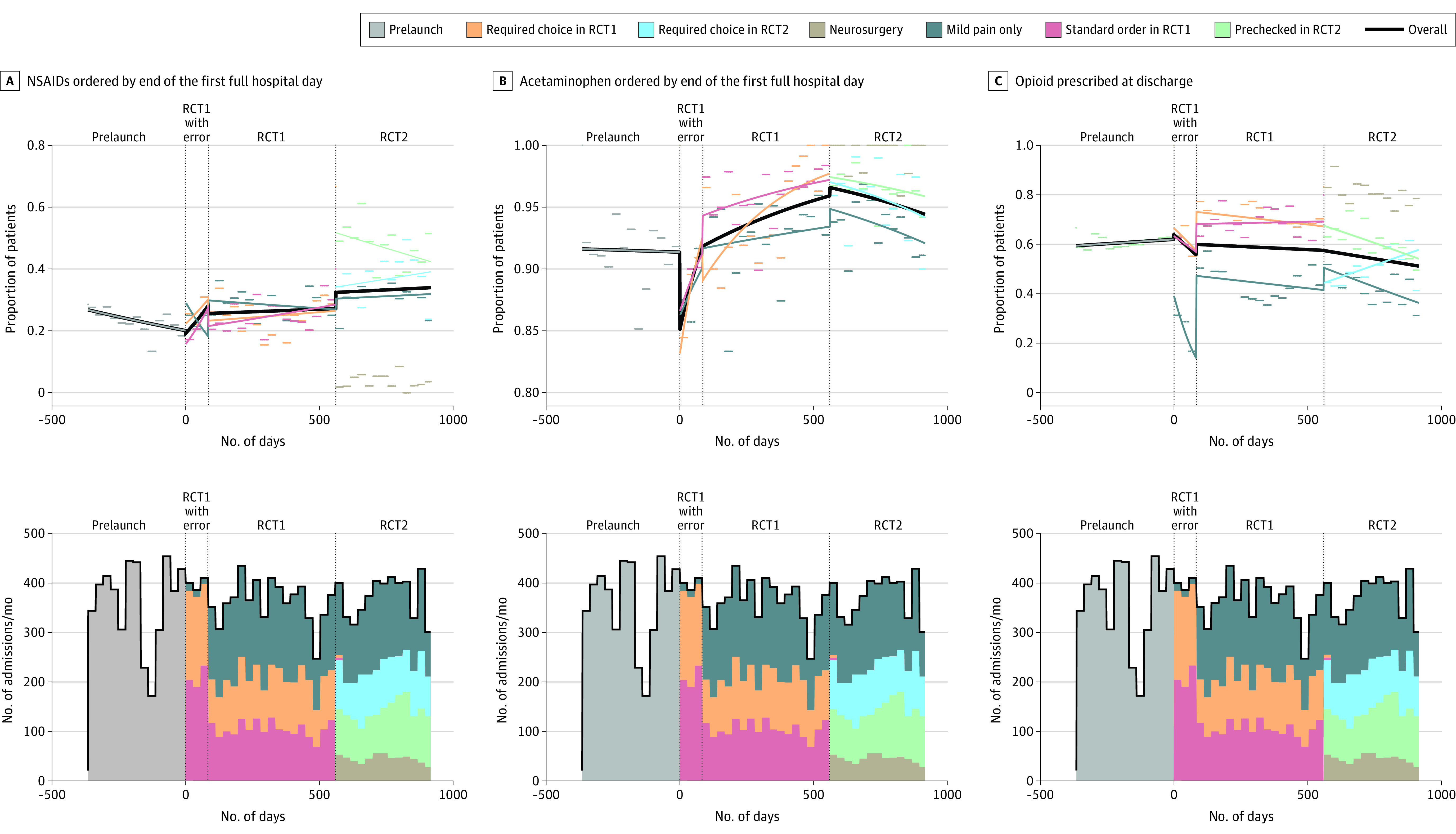
Interrupted Time Series Analysis of Acetaminophen, Nonsteroidal Antiinflammatory Drugs (NSAIDS), and Opioid Orders Over Time Proportions of patients in each month with the given outcome for each group are plotted as short horizontal lines. For each group and during each specified time period, a linear best fit line is plotted using predictions from a logistic regression model with time as the only independent variable. The linear best fit overall, irrespective of group, is also plotted. Vertical lines demarcate periods: period 0 (prelaunch), period 1 (RCT1 launch with error in order set), period 2 (RCT1), and period 3 (RCT2). RCT indicates randomized clinical trial.

## Discussion

We found that prechecking orders at admission for surgical patients for both acetaminophen and NSAIDs increased ordering of these multimodal pain regimens. However, the rate of NSAID use remained low, perhaps representing clinician resistance to its use postoperatively despite studies showing its safety.^5^ Opioid prescribing at discharge appeared to decrease, likely due to multiple concomitant institution-wide campaigns to standardize opioid prescribing. However, the fact that acetaminophen rose in all groups, including the mild pain group, suggests a learning effect from the intervention on clinician ordering overall, not just when orders are prechecked. Importantly, we were able to test and avoid interventions causing more electronic health record work, but this intervention was not ultimately useful. Although our study was conducted at a quaternary care center over 3 distinct hospital sites, generalizability remains limited given it is a single-institution study.

## Notes

Supplement. Data sharing statement
