# Supplementary material for: Optimizing Uptake of Multimodal Pain Management After Surgery Using the Electronic Health Record
Source: JAMA Surg. 2023 Aug 23;158(10):1108–11. doi: 10.1001/jamasurg.2023.3654 (PMC10448375; doi:10.1001/jamasurg.2023.3654)
Supplement: Supplement. — Data sharing statement [file jamasurg-e233654-s001.pdf]

Data Sharing Statement

Bongiovanni. Optimizing Uptake of Multimodal Pain Management After Surgery Using the Electronic Health Record. *JAMA Surg*. Published August 23, 2023. doi:10.1001/jamasurg.2023.3654

Data
Data available: No
